# Data Mining Approach to Melatonin Treatment in Alzheimer’s Disease: New Gene Targets *MMP2* and *NR3C1*

**DOI:** 10.3390/ijms26010338

**Published:** 2025-01-02

**Authors:** Jingyi Zhang, Ka Chun Tsui, Hoi Ying Lee, Luca Aquili, Kah Hui Wong, Ersoy Kocabicak, Yasin Temel, Zhiliang Lu, Man-Lung Fung, Allan Kalueff, Lee Wei Lim

**Affiliations:** 1 Department of Biosciences and Bioinformatics, School of Science, Xi’an Jiaotong-Liverpool University, Suzhou 215123, Chinatsuikc@connect.hku.hk (K.C.T.); luca.aquili@murdoch.edu.au (L.A.); zhiliang.lu@xjtlu.edu.cn (Z.L.); avkalueff@gmail.com (A.K.); 2 Suzhou Municipal Key Laboratory of Neurobiology and Cell Signaling, School of Science, Xi’an Jiaotong-Liverpool University, Suzhou 215123, China; 3 School of Biomedical Sciences, Li Ka Shing Faculty of Medicine, The University of Hong Kong, Hong Kong, China; hoi.lee20@imperial.ac.uk (H.Y.L.); fungml@hku.hk (M.-L.F.); 4 College of Science, Health, Engineering and Education, Discipline of Psychology, Murdoch University, Perth 6150, Australia; 5 Department of Anatomy, Faculty of Medicine, Universiti Malaya, Kuala Lumpur 50603, Malaysia; 6 Atlas University, 34406 Istanbul, Turkey; ersoy.kocabicak@atlas.edu.tr; 7 Department of Neurosurgery, Maastricht University, 6202 Maastricht, The Netherlands; y.temel@maastrichtuniversity.nl; 8 Suzhou Municipal Key Laboratory of Cancer Biology and Chronic Diseases, School of Science, Xi’an Jiaotong-Liverpool University, Suzhou 215123, China

**Keywords:** melatonin, Alzheimer’s disease, data mining, gene targets, GO enrichment, KEGG pathway

## Abstract

Melatonin is a hormone released by the pineal gland that regulates the sleep–wake cycle. It has been widely studied for its therapeutic effects on Alzheimer’s disease (AD), particularly through the amyloidosis, oxidative stress, and neuroinflammation pathways. Nevertheless, the mechanisms through which it exerts its neuroprotective effects in AD are still largely unknown. Data mining was used to identify potential gene targets that link melatonin’s effects to AD pathways, yielding a comprehensive view of the underlying molecular mechanisms. We identified 3397 genes related to AD from DisGeNet and 329 melatonin gene targets from ChEMBL, which revealed 223 overlapping genes and the potential shared pathways. These genes were used to construct a protein–protein interaction (PPI) network comprising 143 nodes and 823 edges, which demonstrated significant PPI enrichment. A cluster analysis highlighted two key clusters centered on *MMP2* and *NR3C1*, with both genes playing crucial roles in steroid hormone signaling, apoptosis, and monoamine neurotransmission. Gene Ontology (GO) enrichment and KEGG pathway analyses further elucidated their involvement in critical pathways, for instance, steroid hormone signaling and apoptosis regulation, significantly influencing AD pathology through mechanisms such as extracellular matrix remodeling, epigenetic modifications, and neuroinflammation. Our findings emphasize *MMP2* and *NR3C1* as important gene targets for future research on melatonin treatment in AD, paving the way for further investigations into their roles in AD pathophysiology.

## 1. Introduction

Alzheimer’s disease (AD) is the most common form of dementia in the world and the fifth leading cause of death among older Americans [1]. AD impairs the central nervous system, including the cerebral cortex, hippocampus, dorsal raphe, and ventral tegmental area [2,3], and causes a progressive decline in cognitive functions, psychological changes, and sleep disorders [4]. Melatonin, secreted by the pineal gland, normally regulates the sleep–wake cycle but is greatly inhibited in patients with AD [5,6,7]. Consequently, melatonin has been prescribed to treat sleep disorders in AD patients. Some studies report that melatonin is also able to inhibit the progression of AD neuropathology and reverse cognitive impairment [8,9], and further investigations in systematic reviews and meta-analyses of randomized controlled trials support its potential as a promising therapeutic approach for improving cognitive decline in mild AD and mild cognitive impairment [9,10,11,12].

Although the precise causes of AD are still unknown, clinical studies suggest the involvement of neuronal degeneration, the accumulation of abnormal protein turnover, disturbed neurotransmission systems/cell–cell communications, and energy metabolism/mitochondrial malfunction [6]. The characteristic hallmarks of AD are the presence of the abnormal accumulation of amyloid-beta (Aβ) plaques and neurofibrillary tangles (NFTs) [13]. In preclinical studies of AD, melatonin was shown to enhance Aβ lymphatic clearance in a transgenic mouse model of amyloidosis [14] and restore neurotransmission functions by regulating acetylcholine [15,16] and glutamate levels [17,18]. However, systematic analyses and explorations of gene–gene or protein–protein interaction networks of melatonin treatment in AD remain elusive.

Advancements in microarray and next-generation sequencing (NGS) technologies have allowed large genomic repositories for data mining, including the UniProt [19], Gene Expression Omnibus (GEO) [20], PubChem [21], and EMBOSS [22] databases, for analyzing differentially expressed genes (DEGs) [23] in various pathogenic conditions [24]. There are also many bioinformatics software tools and algorithms available to identify the gene targets and signaling pathways of melatonin in AD. In this study, we used different databases to mine the genes of interest and then performed a cluster analysis, Gene Ontology (GO) enrichment analysis, and Kyoto Encyclopedia of Genes and Genomes (KEGG) pathway analysis to identify the gene targets and associated pathways of melatonin treatment in AD.

## 2. Results

We identified 3397 genes that were related to AD pathology from DisGeNet and 329 genes that were predicted to be targets of melatonin from ChEMBL. A total of 223 genes were found to be overlapped across both sets of genes. These genes were examined in the subsequent analyses. All genes are listed in Supplementary Table S1.

### 2.1. AD Protein–Protein Interaction (PPI) Network

**Figure 1:**
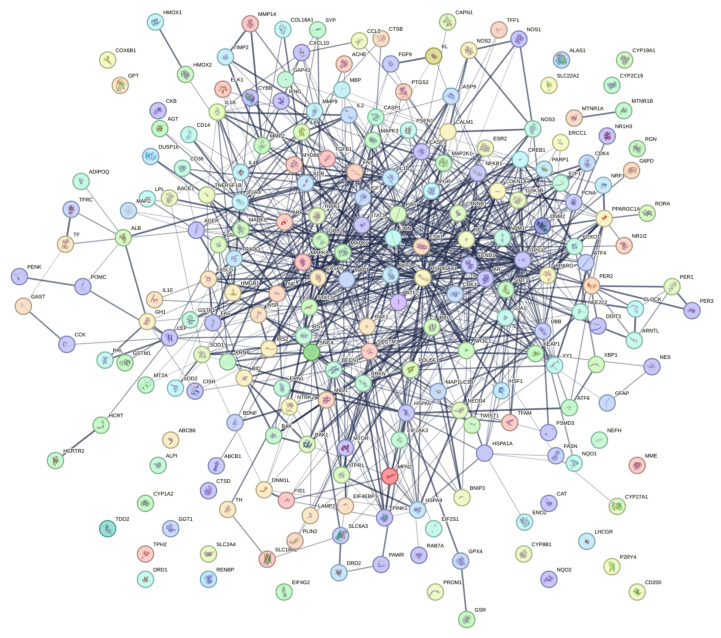
Genes related to AD were retrieved from DisGeNET, and melatonin gene targets were retrieved from ChEMBL. Overlapped gene targets are shown.

The general PPI network of AD protein-encoding genes was constructed (Figure 1). This PPI network had 143 nodes and 823 edges, with an average node degree of 11.4 and PPI enrichment *p*-value of <1.0 × 10^−16^. The clustered genes in the center with surrounding dense edges were considered to be promising therapeutic targets. These candidate genes included *CREB*, *MET*, *ERBB2*, *AR*, *MME*, *MMP2*, *IKBKB*, *HDAC1*, *PIL3R1*, *GSK3B*, *MAPK4*, *ESR1*, *PGR*, *ITGB1*, *CAP3*, *ATM*, and *NR3C1*. On the bottom right of the network, we also observed a second cluster of genes with dense edges, which included *SLC6A4*, *SLC6A3*, *CHRNA3*, *DRD2*, etc.

### 2.2. Cluster Analysis

**Figure 2:**
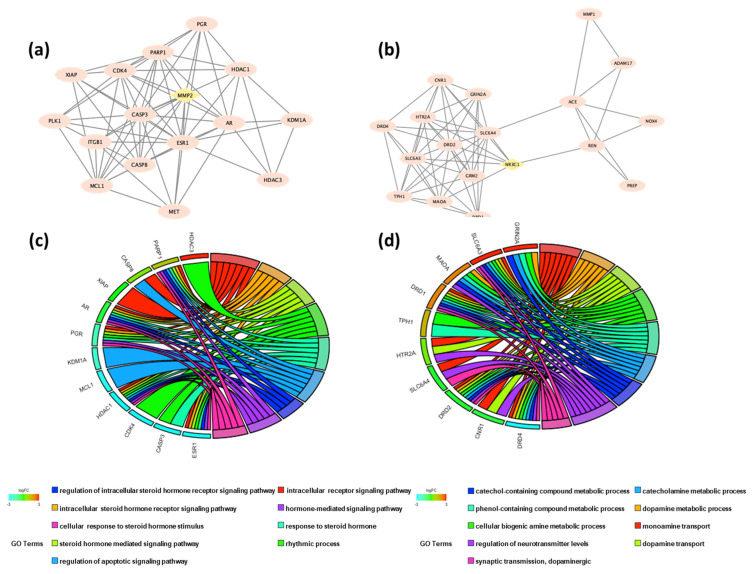
Cluster analysis of melatonin–AD overlapping genes. Cluster 1 network (**a**) and Cluster 2 network (**b**) depict the protein–protein interactions within each cluster of overlapping genes. Chord diagrams of Gene Ontology (GO) analyses show enriched biological processes for Cluster 1 (**c**) and Cluster 2 (**d**). Chord diagrams display connections between enriched GO terms and genes involved in each process, illustrating functional links within clusters.

The melatonin–AD overlapping genes formed two clusters with K-core set to 6. Cluster 1 contained 16 genes, and Cluster 2 contained 18 genes (Figure 2a,b). Detailed information on each cluster and the corresponding gene lists are shown in Table 1.

Cluster 1, with 16 nodes and 68 edges, had a score of 9.067, indicating its robustness and significance, with *MMP2* as the key seed node (Figure 2a). The *MMP2* gene is located on chromosome 16 at position 12.2 and encodes for matrix metallopeptidase 2 (MMP2), a type IV collagenase that is involved in the breakdown of extracellular matrix (ECM) in normal physiological processes and acts as a novel H3NT protease possibly related to epigenetic modifications in AD [25,26]. Cluster 2 had 18 nodes and 60 edges, with a score of 7.059 (Figure 2b). The seed node in Cluster 2 was *NR3C1*, which is a gene located on chromosome 5. The *NR3C1* gene encodes for glucocorticoid receptor, which is involved in upregulating the expression of anti-inflammatory proteins in the nucleus and downregulating proinflammatory proteins in the cytosol [27,28].

### 2.3. GO Enrichment Analysis

**Figure 3:**
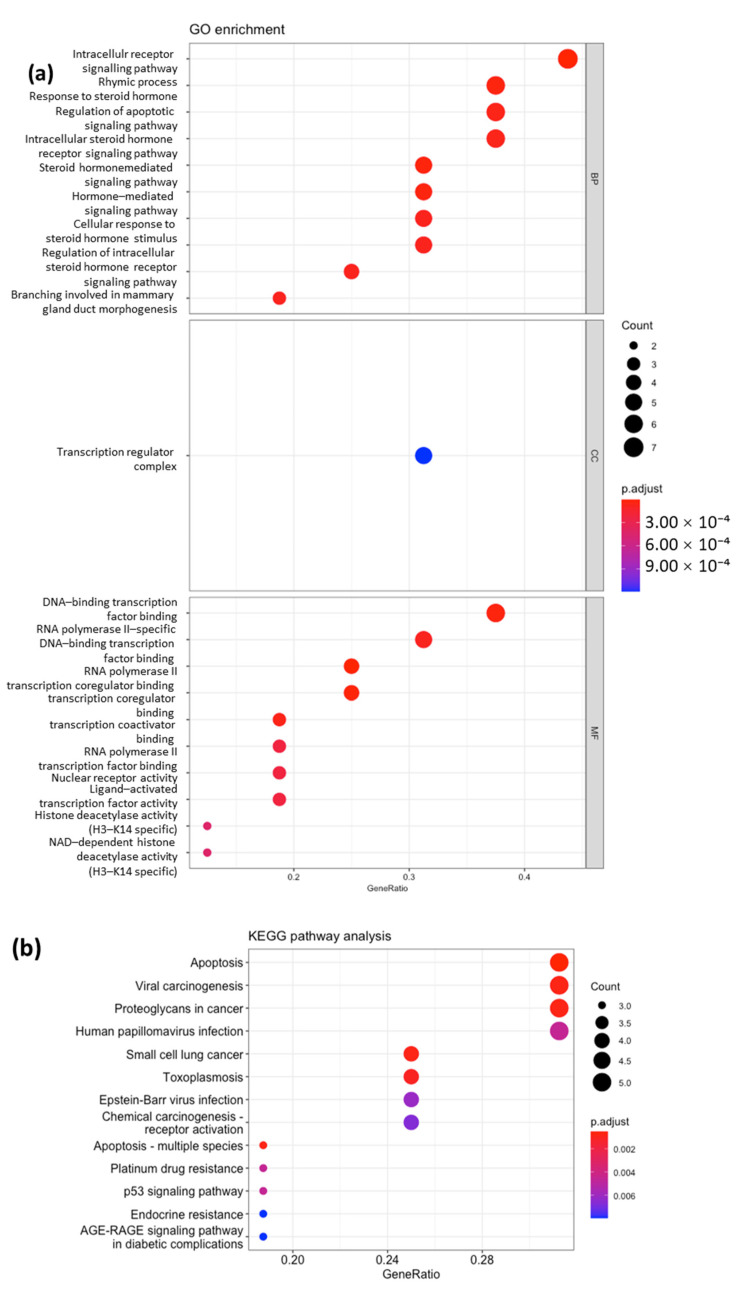
Bubble maps of GO annotation analysis (**a**) and KEGG pathway analysis (**b**) for Cluster 1. The Y-axis lists enriched GO terms and KEGG pathway names. Each bubble’s area represents the number of genes associated with a term, while the bubble color reflects the adjusted p-value significance, with blue indicating low significance and red indicating high significance.

**Figure 4:**
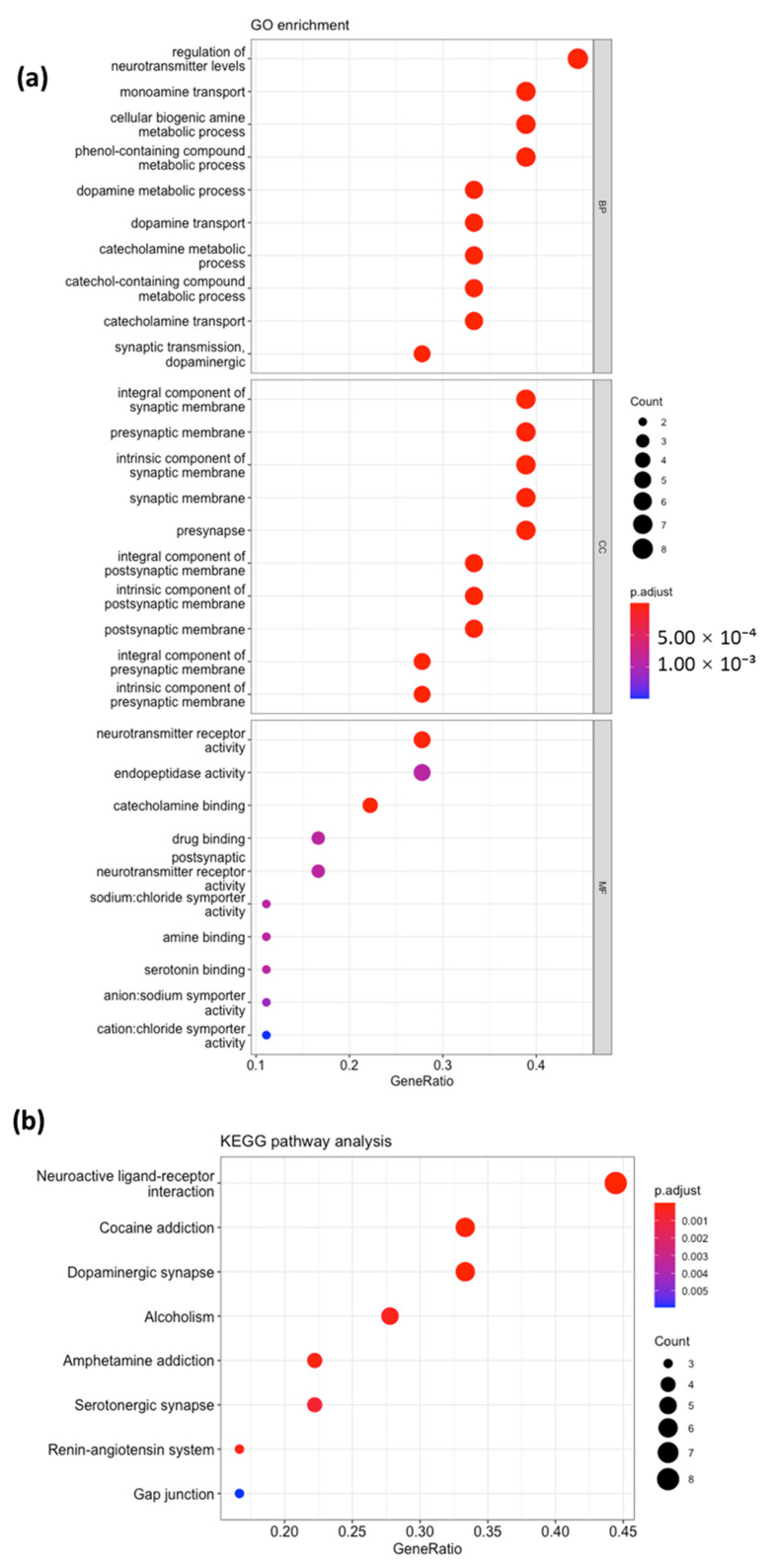
Bubble maps of GO annotation analysis (**a**) and KEGG pathway analysis (**b**) for Cluster 2. The Y-axis lists enriched GO terms and KEGG pathway names. Each bubble’s area represents the number of genes associated with a term, while the bubble color reflects the adjusted *p*-value significance, with blue indicating low significance and red indicating high significance.

To further analyze the two clusters, we performed a GO enrichment analysis. In Cluster 1, we found 140 biological process (BP) GO terms, 1 GO term related to the cellular component (CC), and 41 GO terms related to molecular function (MF). The top 10 GO terms were visualized in Cluster 1 (Figure 3a) and Cluster 2 (Figure 4a). The connectivity of each gene to the top 10 BPs in Cluster 1 and Cluster 2 is shown in Figure 2c,d. The top 40 BP GO terms in Cluster 1 (Table 2) and Cluster 2 (Table 3) were also examined for a more comprehensive analysis.

Among the top 10 BP GO terms in Cluster 1, the most significantly enriched processes were related to steroid hormone signaling and apoptotic signaling pathways (Figure 3a). The most frequent genes in these pathways included *AR*, *XIAP*, *ES1*, *CASP8*, *PARP1*, *PGR*, and *HDAC1*. However, the seed node of Cluster 1, *MMP2*, was not observed in the top 10 BP GO terms. *MMP*2 was found in the mid-20 to mid–late 30 pathways related to cellular responses to UV, abiotic stimuli, environmental stimuli, and chemical stress, and female pregnancy. Among the top 10 BP GO terms in Cluster 2, the most significantly enriched processes were related to monoamine neurotransmission, such as dopamine and catecholamine. The most frequent genes in these pathways included *SLC6A4*, *DRD4*, *DRD2*, *DRD1*, *SLC6A3*, *HTR2A*, *CNR1*, *TPH1*, etc. The detailed gene connectivity to each biological process is shown in Figure 2d. However, the seed node of Cluster 2, *NR3C1*, was not observed even in the top 40 BP GO terms and was not found in any glucocorticoid pathways in Cluster 2. *NR3C1*, a glucocorticoid receptor, regulates stress response genes, while *MMP2*, an enzyme involved in extracellular matrix remodeling, plays a key role in tissue repair, highlighting their interplay in cellular stress and injury responses.

### 2.4. KEGG Pathway Analysis

**Figure 5:**
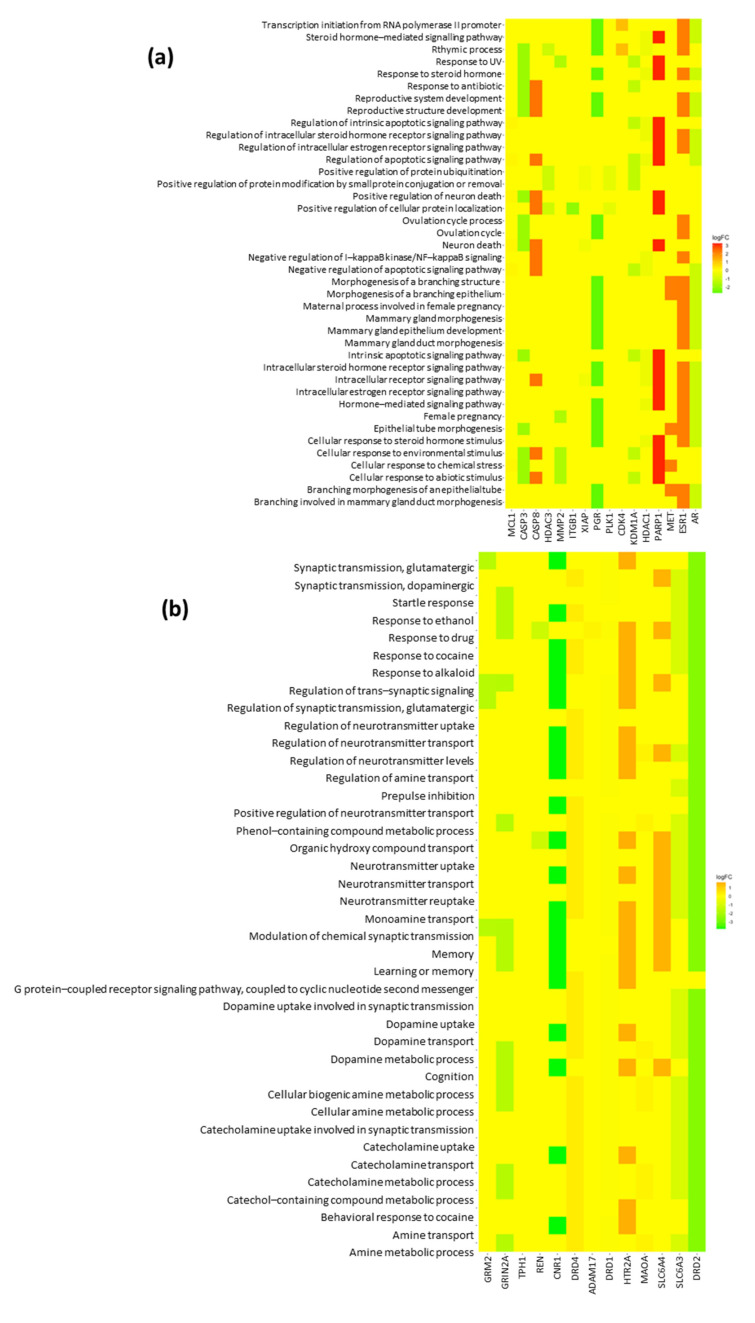
Heatmaps showing enriched biological processes for Cluster 1 (**a**) and Cluster 2 (**b**). Rows represent enriched biological processes, and columns represent genes within each cluster. Color intensity reflects gene association levels with each process, aiding in the visualization of process-specific gene expression patterns.

The KEGG pathway analysis identified 13 pathways in Cluster 1 (Figure 3b) and 8 pathways in Cluster 2 (Figure 4b). An analysis of the results showed that Cluster 1 genes were associated with apoptosis, whereas Cluster 2 genes were associated with monoamine neurotransmission, particularly serotonin and dopamine (Figure 5).

## 3. Discussion

By integrating the cluster analysis with GO and KEGG analyses, we identified that Cluster 1, with its *MMP2* seed node, mainly involved genes related to ECM breakdown and epigenetic modifications in AD, whereas Cluster 2, with its *NR3C1* seed node, mainly involved genes related to the regulation of glucocorticoid receptors. However, both *MMP2* and *NR3C1* were not in the top 10 BP GO terms of their respective clusters, and *NR3C1* was not even in the top 40 BP GO terms. This result suggests that these two gene targets have never been studied in association with melatonin treatment in AD, despite several studies reporting the potential role of *MMP2* and *NR3C1* in AD [29,30].

*MMP2* plays a vital role in tissue remodeling and turnover by specifically targeting and degrading proteins such as collagen and fibronectin [31]. *MMP2* also plays a crucial role in epigenetic modifications related to AD by facilitating myogenic gene activation and Aβ degradation [25], as demonstrated by the effects of novel HDAC inhibitors that target *MMP2* activity [32]. Melatonin is primarily synthesized and secreted by the pineal gland in the brain and regulates circadian rhythms [33], sleep–wake cycles [34], and various physiological processes [35]. Melatonin has also been shown to regulate the expression of *MMP2*, mediated by SIRT1, a key enzyme involved in various cellular processes [36,37]. Melatonin can inhibit SIRT1 to modulate *MMP2* activity, which was shown to impact the degradation of the ECM and exert anti-proliferative effects on prostate tumor cells [36,38]. Moreover, *MMP2* has been linked to the regulation of neurotransmission [39] and is also involved in the remodeling of synapses via the degradation and turnover of ECM components within the synaptic environment [40,41]. Consequently, *MMP2* is involved in regulating the structural plasticity and functionality of synapses, and it plays a crucial role in shaping the efficiency and effectiveness of neurotransmission processes.

*NR3C1* functions as a transcription factor by modulating the expression of target genes upon binding with glucocorticoids [27]. Notably, *NR3C1* interacts with melatonin receptors [42,43], suggesting potential cross-regulation between glucocorticoid and melatonin signaling pathways [44]. Although the precise mechanisms and implications of this interaction are still under investigation, this underscores *NR3C1*′s potential role in mediating the effects of melatonin in various cellular processes [43,45]. Additionally, *NR3C1* enhances *DRD2* expression in the miR-124-1+/− prefrontal cortex in mice, indicating its critical role in modulating neurotransmitter pathways and PFC function [46]. *NR3C1* is expressed in various brain regions and is essential for regulating neurotransmission and neuronal circuit activity [47,48]; its dysregulation is linked to altered neurotransmitter systems, impaired synaptic plasticity, and increased susceptibility to neuroinflammation and oxidative stress [49,50]. Such impairments can significantly impact the overall efficiency and integrity of neurotransmission processes, potentially contributing to the development of neurological disorders such as AD. Increased *NR3C1* activation has been shown to cause neuronal damage and cognitive decline [51], modulate Aβ metabolism, and influence neuroinflammation [52], further linking it to AD pathogenesis. Epigenetic modifications, such as DNA methylation in the *NR3C1*, have also been associated with altered hypothalamic–pituitary–adrenal axis function [53,54] and increased stress vulnerability [55], both of which may heighten the risk of AD. These findings highlight *NR3C1* as a critical factor in understanding AD and underscore its potential as a therapeutic target.

*MMP2* and *NR3C1* have been identified to have significant associations with melatonin, and they were shown to be differentially expressed in AD in a preclinical study. Increased levels of *MMP2* have been observed in postmortem AD brains [56] and in the cerebrospinal fluid of AD patients [57], indicating its potential as a biomarker for disease progression. Animal models of AD frequently exhibit altered *MMP2* expression, highlighting its connection to Aβ pathology and blood–brain barrier disruption [58]. Furthermore, *MMP2* plays a crucial role in Aβ clearance, neuroinflammation, and synaptic plasticity [59], making it a vital factor in maintaining neuronal health. In AD, *MMP2* was shown to degrade Aβ via cleaving soluble Aβ peptides, was upregulated around astrocytes in AD brain [60], and was shown to accumulate near NFTs to eliminate the toxic form of tau [61]. Melatonin supplementation has been reported to be effective in increasing *MMP2* activity in gastric ulceration [23] and decreasing *MMP2* expression in cancer stem cells and SKOV3 cells [62]. However, the effect of melatonin treatment on *MMP2* expression in AD has never been studied. The GO enrichment analysis suggested that Cluster 1 was enriched in steroid hormone signaling and apoptotic signaling pathways. *MMP2* has been found to be associated with changes in steroid hormones in endometriosis sera and peritoneal fluid [63].

According to the results of the top 40 BP GO terms, *MMP2* is also associated with female pregnancy. This might indicate the possible mechanism of melatonin treatment in AD via manipulating estrogen and progesterone levels. Existing studies have found that melatonin can inhibit estrogen receptor transactivation in breast cancer stem cells [64] and induce progesterone production in human granulosa lutein cells [65]. Estrogen and selective estrogen receptor modulators have both beneficial and harmful roles in AD [66]. On the other hand, progesterone has been found to have neuroprotective effects [67]. These lines of evidence highlight the need to further investigate the target genes identified in the cluster analysis.

In Cluster 2, the GO enrichment analysis also indicated that melatonin can interfere with dopaminergic and catecholaminergic neurotransmission. Dopamine was found to be downregulated in the hippocampus and VTA and upregulated in the frontal cortex and SNR networks in a mouse model of AD [6]. Melatonin was also shown to reduce the dopamine content in the neuro-intermediate lobe of male hamsters [68]. Melatonin was also able to suppress catecholamine synthesis by inhibiting the MT1-cAMP pathway in adrenomedullary cells [44] and promoting Smad signaling [44]. However, the molecular mechanism of how melatonin regulates these two monoamine neurotransmission pathways remains unclear. Notably, the seed node *NR3C1* in Cluster 2 was not observed in any pathway of the top 40 BP GO terms. *NR3C1* encodes glucocorticoid receptors, which are widely expressed in the brain. Glucocorticoids and corticotropin-related hormones can induce AD-associated pathologies [69]. Although melatonin has been found to regulate glucocorticoid receptors [70], whether there is melatonin and glucocorticoid receptor crosstalk in AD is currently not known. Based on the GO results, the involvement of glucocorticoid receptors in monoamine neurotransmission signaling pathways in AD should be further investigated.

Melatonin can help mitigate AD symptoms by enhancing neurotransmitter balance and reducing neuroinflammation [6,35], both of which are crucial for maintaining cognitive function. Specifically, melatonin could influence dopaminergic and catecholaminergic neurotransmission, potentially addressing behavioral and mood-related aspects of AD [71,72]. Moreover, the modulation of stress-related hormones by *NR3C1* could further protect neuronal health and support cognitive resilience [73,74]. Also, we should acknowledge that the STRING network likely treats interactions as binary (present/absent), which limits its ability to capture the variability in interaction strength, context, and biological relevance, highlighting the need for future analyses to incorporate models that reflect these complexities. Further experimental and clinical research is needed to validate these interactions to ultimately clarify the therapeutic potential of melatonin in AD.

In summary, we identified a total of 3397 genes related to AD from DisGeNet and 329 melatonin target genes from ChEMBL, revealing an overlap of 223 genes. A cluster analysis highlighted two key clusters centered on the *MMP2* and *NR3C1* genes, which both play crucial roles in steroid hormone signaling, apoptosis, and monoamine neurotransmission. Our findings provide important gene targets for future research on melatonin treatment in AD, paving the way for further investigations into their roles in AD pathophysiology.

## 4. Methods and Materials

### 4.1. Database Mining for AD-Related Genes and Melatonin Gene Targets

Genes related to AD were retrieved from DisGeNET (https://www.disgenet.org; accessed on 15 June 2021), a publicly available database of human disease-related genes curated from Genome-Wide Association Studies (GWASs) and the scientific literature [75]. Gene targets of melatonin were obtained from the ChEMBL database, a database that includes drug, chemical, bioactivity, and genomic data. ChEMBL also has a unique target prediction function that uses quantitative structure–activity relationship (QSAR) models and conformal predictors to predict the gene target of a drug of interest [76]. DisGeNET was chosen for its comprehensive gene–disease associations, while ChEMBL provides detailed data on bioactive compounds, offering insights that are not as effectively covered by UniProt and GeneCards.

### 4.2. Protein–Protein Interaction (PPI) Networks of Alzheimer’s Disease

We retrieved all genes related to AD and then used STRING to study the functional associations between proteins. STRING is a resource that can predict protein–protein or functional protein-encoding gene interactions [77]. The network was generated based on “homo sapiens” using a minimum required interaction score cutoff of 0.4 to balance the inclusion of relevant interactions while minimizing false positives, ensuring moderate to high confidence in the results. After network generation, the genes in the center with dense surrounding edges represented the gene hallmarks of AD and potential therapeutic targets. These genes are integral to critical pathways regulating cellular functions. The interconnections imply that targeting a central gene could yield broader therapeutic effects by influencing multiple related pathways in AD. Additionally, central genes often play pivotal roles in disease mechanisms, enhancing their potential as therapeutic targets. Their evolutionary conservation further underscores their importance in understanding and addressing AD.

### 4.3. Gene Cluster Analysis

We performed a simple manual comparison and filtering of the AD gene list and melatonin gene targets, identifying overlapping genes. Cytoscape 3.8.2 was used to segregate the overlapping genes into clusters with the K-core cutoff set at 6; this was chosen to ensure that only the most interconnected genes were included, thereby highlighting the core components of the network that are likely to play critical roles in the biological processes under investigation. Each cluster represents a sub-set with the highest connectivity in terms of their involvement in the same biological processes or the same specific functions [78,79]. The results were further processed by the MCODE module in Cytoscape to analyze clusters in the network, utilizing the default parameters to ensure a standardized approach in identifying the most densely connected regions of the network.

### 4.4. GO Enrichment Analysis and KEGG Analysis

A Gene Ontology (GO) enrichment analysis and Kyoto Encyclopedia of Genes and Genomes (KEGG) pathway analysis were performed separately on each cluster in R 4.1.1 version. The GO analyses utilized the clusterProfiler package 4.14.0 version. The *p*-value thresholds of ≥0.01 for the top 15 results of the GO term components and the top 20 KEGG pathways were optimized based on preliminary analyses. We also selected the top 40 results for each cluster to avoid missing any important findings. Finally, we compared the GO and KEGG results with the cluster analysis results and AD PPI results to reveal new gene targets and pathways for future study.

## Figures and Tables

**Table 1:** Clusters of the AD–melatonin PPI network. This table presents the clusters identified in the AD–melatonin PPI network, along with their corresponding scores, numbers of nodes and edges, and associated gene symbols.

Cluster	Score	Nodes	Edges	Gene Symbols
1	9.067	16	68	*ESR1*, *MMP2*, *AR*, *PGR*, *KDM1A*, *MCL1*, *ITGB1*, *MET*, *HDAC1*, *CASP3*, *XIAP*, *CDK4*, *HDAC3*, *PLK1*, *PARP1*, *CASP8*
2	7.059	18	60	*DRD4*, *SLC6A4*, *GRM2*, *NR3C1*, *ACE*, *MMP1*, *HTR2A*, *REN*, *DRD2*, *MAOA*, *GRIN2A*, *PREP*, *DRD1*, *CNR1*, *TPH1*, *ADAM17*, *NOX4*, *SLC6A3*

**Table 2:** Top GO biological process terms for Cluster 1. This table lists the top GO biological process terms associated with Cluster 1, including their respective IDs, the numbers of genes involved, and the adjusted p-values indicating the statistical significance of each term.

ID	GO Term	Genes	Adj. *p*-Value
GO:0030522	intracellular receptor signaling pathway	*AR*, *XIAP*, *ESR1*, *CASP8*, *PARP1*, *PGR*, *HDAC1*	1.65 × 10⁻⁶
GO:0030518	intracellular steroid hormone receptor signaling pathway	*AR*, *ESR1*, *PARP1*, *PGR*, *HDAC1*	1.86 × 10⁻⁵
GO:0043401	steroid hormone-mediated signaling pathway	*AR*, *ESR1*, *PARP1*, *PGR*, *HDAC1*	2.92 × 10⁻⁵
GO:0048511	rhythmic process	*CDK4*, *HDAC3*, *CASP3*, *ESR1*, *PGR*, *HDAC1*	3.05 × 10⁻⁵
GO:0048545	response to steroid hormone	*AR*, *CASP3*, *ESR1*, *PARP1*, *PGR*, *HDAC1*	4.82 × 10⁻⁵
GO:2001233	regulation of apoptotic signaling pathway	*AR*, *CASP8*, *PARP1*, *MCL1*, *KDM1A*, *HDAC1*	5.50 × 10⁻⁵
GO:0033143	regulation of intracellular steroid hormone receptor signaling pathway	*AR*, *ESR1*, *PARP1*, *HDAC1*	6.60 × 10⁻⁵
GO:0009755	hormone-mediated signaling pathway	*AR*, *ESR1*, *PARP1*, *PGR*, *HDAC1*	6.76 × 10⁻⁵
GO:0071383	cellular response to steroid hormone stimulus	*AR*, *ESR1*, *PARP1*, *PGR*, *HDAC1*	7.21 × 10⁻⁵
GO:0060444	branching involved in mammary gland duct morphogenesis	*AR*, *ESR1*, *PGR*	7.21 × 10⁻⁵
GO:2001234	negative regulation of apoptotic signaling pathway	*AR*, *CASP8*, *MCL1*, *KDM1A*, *HDAC1*	9.79 × 10⁻⁵
GO:1901216	positive regulation of neuron death	*CASP3*, *CASP8*, *PARP1*, *MCL1*	9.79 × 10⁻⁵
GO:0060603	mammary gland duct morphogenesis	*AR*, *ESR1*, *PGR*	1.59 × 10⁻⁴
GO:0031398	positive regulation of protein ubiquitination	*PLK1*, *HDAC3*, *XIAP*, *KDM1A*	2.27 × 10⁻⁴
GO:0097193	intrinsic apoptotic signaling pathway	*CASP3*, *PARP1*, *MCL1*, *KDM1A*, *HDAC1*	2.38 × 10⁻⁴
GO:1903829	positive regulation of cellular protein localization	*PLK1*, *ITGB1*, *HDAC3*, *CASP8*, *PARP1*	2.73 × 10⁻⁴
GO:0060562	epithelial tube morphogenesis	*MET*, *AR*, *CASP3*, *ESR1*, *PGR*	2.97 × 10⁻⁴
GO:0033146	regulation of intracellular estrogen receptor signaling pathway	*AR*, *ESR1*, *PARP1*	2.97 × 10⁻⁴
GO1903322	positive regulation of protein modification by small protein conjugation or removal	*PLK1*, *HDAC3*, *XIAP*, *KDM1A*	2.97 × 10⁻⁴
GO:0060443	mammary gland morphogenesis	*AR*, *ESR1*, *PGR*	2.97 × 10⁻⁴
GO:0048754	branching morphogenesis of an epithelial tube	*MET*, *AR*, *ESR1*, *PGR*	2.97 × 10⁻⁴
GO:0009411	response to UV	*MMP2*, *CASP3*, *PARP1*, *KDM1A*	2.97 × 10⁻⁴
GO:0071214	cellular response to abiotic stimulus	*MMP2*, *CASP3*, *CASP8*, *PARP1*, *KDM1A*	2.97 × 10⁻⁴
GO0104004	cellular response to environmental stimulus	*MMP2*, *CASP3*, *CASP8*, *PARP1*, *KDM1A*	2.97 × 10⁻⁴
GO:0022602	ovulation cycle process	*CASP3*, *ESR1*, *PGR*	2.97 × 10⁻⁴
GO:0046677	response to antibiotic	*CASP3*, *CASP8*, *KDM1A*	2.97 × 10⁻⁴
GO:0070997	neuron death	*XIAP*, *CASP3*, *CASP8*, *PARP1*, *MCL1*	3.33 × 10⁻⁴
GO:0062197	cellular response to chemical stress	*MMP2*, *MET*, *CASP3*, *PARP1*, *MCL1*	3.44 × 10⁻⁴
GO:2001242	regulation of intrinsic apoptotic signaling pathway	*PARP1*, *MCL1*, *KDM1A*, *HDAC1*	3.68 × 10⁻⁴
GO:0043124	negative regulation of I-kappaB kinase/NF-kappaB signaling	*ESR1*, *CASP8*, *HDAC1*	3.83 × 10⁰
GO:0061138	morphogenesis of a branching epithelium	*MET*, *AR*, *ESR1*, *PGR*	4.69 × 10⁻⁴
GO:0007565	female pregnancy	*MMP2*, *AR*, *ESR1*, *PGR*	4.97 × 10⁻⁴
GO:0060135	maternal process involved in female pregnancy	*AR*, *ESR1*, *PGR*	5.21 × 10⁻⁴
GO:0030520	intracellular estrogen receptor signaling pathway	*AR*, *ESR1*, *PARP1*	5.34 × 10⁻⁴
GO:0001763	morphogenesis of a branching structure	*MET*, *AR*, *ESR1*, *PGR*	5.49 × 10⁻⁴
GO:0006367	transcription initiation from RNA polymerase II promoter	*CDK4*, *AR*, *ESR1*, *PGR*	5.49 × 10⁻⁴
GO:0048608	reproductive structure development	*AR*, *CASP3*, *ESR1*, *CASP8*, *PGR*	5.51 × 10⁻⁴
GO:0042698	ovulation cycle	*CASP3*, *ESR1*, *PGR*	6.00 × 10⁻⁴
GO:0061180	mammary gland epithelium development	*AR*, *ESR1*, *PGR*	6.14 × 10⁻⁴

**Table 3:** Top GO biological process terms for Cluster 2. This table lists the top GO biological process terms associated with Cluster 2, including their respective IDs, the numbers of genes involved, and the adjusted *p*-values indicating the statistical significance of each term.

ID	GO Term	Genes	Adj. *p*-Value
GO:0015844	monoamine transport	*SLC6A4*, *DRD4*, *DRD2*, *DRD1*, *SLC6A3*, *HTR2A*, *CNR1*	3.90 × 10⁻¹⁰
GO:0042417	dopamine metabolic process	*GRIN2A*, *DRD4*, *DRD2*, *DRD1*, *MAOA*, *SLC6A3*	3.90 × 10⁻¹⁰
GO:0015872	dopamine transport	*DRD4*, *DRD2*, *DRD1*, *SLC6A3*, *HTR2A*, *CNR1*	8.16 × 10⁻¹⁰
GO:0006576	cellular biogenic amine metabolic process	*GRIN2A*, *DRD4*, *DRD2*, *DRD1*, *MAOA*, *SLC6A3*, *TPH1*	8.16 × 10⁻¹⁰
GO:0018958	phenol-containing compound metabolic process	*GRIN2A*, *DRD4*, *DRD2*, *DRD1*, *MAOA*, *SLC6A3*, *TPH1*	8.16 × 10⁻¹⁰
GO:0006584	catecholamine metabolic process	*GRIN2A*, *DRD4*, *DRD2*, *DRD1*, *MAOA*, *SLC6A3*	8.16 × 10⁻¹⁰
GO:0009712	catechol-containing compound metabolic process	*GRIN2A*, *DRD4*, *DRD2*, *DRD1*, *MAOA*, *SLC6A3*	8.16 × 10⁻¹⁰
GO:0001505	regulation of neurotransmitter levels	*SLC6A4*, *DRD4*, *DRD2*, *DRD1*, *MAOA*, *SLC6A3*, *HTR2A*, *CNR1*	1.03 × 10⁻⁹
GO:0042493	response to drug	*GRIN2A*, *SLC6A4*, *REN*, *DRD2*, *DRD1*, *SLC6A3*, *HTR2A*, *ADAM17*	2.86 × 10⁻⁸
GO:0007613	memory	*GRIN2A*, *SLC6A4*, *DRD2*, *HTR2A*, *CNR1*	1.40 × 10⁻⁶
GO:0050804	modulation of chemical synaptic transmission	*GRIN2A*, *SLC6A4*, *DRD2*, *GRM2*, *DRD1*, *HTR2A*, *CNR1*	1.60 × 10⁻⁶
GO:0099177	regulation of trans-synaptic signaling	*GRIN2A*, *SLC6A4*, *DRD2*, *GRM2*, *DRD1*, *HTR2A*, *CNR1*	1.60 × 10⁻⁶
GO:0007611	learning or memory	*GRIN2A*, *SLC6A4*, *DRD2*, *DRD1*, *HTR2A*, *CNR1*	1.69 × 10⁻⁶
GO:0050890	cognition	*GRIN2A*, *SLC6A4*, *DRD2*, *DRD1*, *HTR2A*, *CNR1*	4.01 × 10⁻⁶
GO:0045471	response to ethanol	*GRIN2A*, *DRD4*, *DRD2*, *SLC6A3*, *CNR1*	2.80 × 10⁻⁶
GO:0006576	cellular biogenic amine metabolic process	*GRIN2A*, *DRD4*, *DRD2*, *DRD1*, *MAOA*, *SLC6A3*, *TPH1*	8.16 × 10⁻¹⁰
GO:0018958	phenol-containing compound metabolic process	*GRIN2A*, *DRD4*, *DRD2*, *DRD1*, *MAOA*, *SLC6A3*, *TPH1*	8.16 × 10⁻¹⁰
GO:0044106	cellular amine metabolic process	*GRIN2A*, *DRD4*, *DRD2*, *DRD1*, *MAOA*, *SLC6A3*, *TPH1*	5.56 × 10⁻⁹
GO:0009308	amine metabolic process	*GRIN2A*, *DRD4*, *DRD2*, *DRD1*, *MAOA*, *SLC6A3*, *TPH1*	6.43 × 10⁻⁹
GO:0042417	dopamine metabolic process	*GRIN2A*, *DRD4*, *DRD2*, *DRD1*, *MAOA*, *SLC6A3*	3.90 × 10⁻¹⁰
GO:0006584	catecholamine metabolic process	*GRIN2A*, *DRD4*, *DRD2*, *DRD1*, *MAOA*, *SLC6A3*	8.16 × 10⁻¹⁰
GO:0009712	catechol-containing compound metabolic process	*GRIN2A*, *DRD4*, *DRD2*, *DRD1*, *MAOA*, *SLC6A3*	8.16 × 10⁻¹⁰
GO:0001964	startle response	*GRIN2A*, *DRD2*, *DRD1*, *SLC6A3*	2.05 × 10⁻⁷
GO:0001964	response to cocaine	*DRD4*, *DRD2*, *SLC6A3*, *HTR2A*, *CNR1*	2.18 × 10⁻⁸
GO:0043279	response to alkaloid	*DRD4*, *DRD2*, *SLC6A3*, *HTR2A*, *CNR1*	9.76 × 10⁻⁷
GO:0048148	behavioral response to cocaine	*DRD4*, *DRD2*, *HTR2A*	3.32 × 10⁻⁶
GO:0015872	dopamine transport	*DRD4*, *DRD2*, *DRD1*, *SLC6A3*, *HTR2A*, *CNR1*	8.16 × 10⁻¹⁰
GO:0051937	catecholamine transport	*DRD4*, *DRD2*, *DRD1*, *SLC6A3*, *HTR2A*, *CNR1*	3.05 × 10⁻⁹
GO:0051583	dopamine uptake involved in synaptic transmission	*DRD4*, *DRD2*, *DRD1*, *SLC6A3*	1.63 × 10⁻⁸
GO:0051934	catecholamine uptake involved in synaptic transmission	*DRD4*, *DRD2*, *DRD1*, *SLC6A3*	1.63 × 10⁻⁸
GO:0090494	dopamine uptake	*DRD4*, *DRD2*, *DRD1*, *SLC6A3*	6.32 × 10⁻⁸
GO:0090493	catecholamine uptake	*DRD4*, *DRD2*, *DRD1*, *SLC6A3*	7.76 × 10⁻⁸
GO:0051952	regulation of amine transport	*DRD4*, *DRD2*, *DRD1*, *HTR2A*, *CNR1*	5.55 × 10⁻⁷
GO:0015837	amine transport	*DRD4*, *DRD2*, *DRD1*, *HTR2A*, *CNR1*	8.06 × 10⁻⁷
GO:0051588	regulation of neurotransmitter transport	*DRD4*, *DRD2*, *DRD1*, *HTR2A*, *CNR1*	9.76 × 10⁻⁷
GO:0051580	regulation of neurotransmitter uptake	*DRD4*, *DRD2*, *DRD1*	1.03 × 10⁻⁵
GO:0051590	positive regulation of neurotransmitter transport	*DRD4*, *DRD2*, *CNR1*	2.69 × 10⁻⁵
GO:0007187	G protein-coupled receptor signaling pathway, coupled to cyclic nucleotide second messenger	*DRD4*, *DRD1*, *HTR2A*, *CNR1*	1.06 × 10⁻⁵
GO:0051966	regulation of synaptic transmission, glutamatergic	*DRD2*, *GRM2*, *DRD1*, *HTR2A*, *CNR1*	1.97 × 10⁻⁷
GO:0035249	synaptic transmission, glutamatergic	*DRD2*, *GRM2*, *DRD1*, *HTR2A*, *CNR1*	6.72 × 10⁻⁷
GO:0060134	prepulse inhibition	*DRD2*, *DRD1*, *SLC6A3*	4.18 × 10⁻⁶

## Data Availability

The data presented in this study are available on request from the corresponding author.

## Supplementary Materials

The following supporting information can be downloaded at: https://www.mdpi.com/article/10.3390/ijms26010338/s1.
